# Supplementary material for: Co-developing a survey on public understanding of sustainable clinical research: A study protocol
Source: PLoS One. 2026 May 11;21(5):e0342279. doi: 10.1371/journal.pone.0342279 (PMC13160332; doi:10.1371/journal.pone.0342279)
Supplement: S1 File — (PDF) [file pone.0342279.s001.pdf]

Public and Patient Involvement (PPI) Information Leaflet

What is this research about?

This research is about public understanding of clinical research. This includes environmental concerns that can be associated with carrying out clinical research. The research for this study will inform aspects of an overall PhD research project investigating sustainable clinical research. An important aspect of this research is the co-development of a public survey.

Why I am doing this research?

This study is part of my PhD research project which is about sustainable clinical research. I want to learn more about public understanding of clinical research, including carrying out environmentally friendly research. As part of this research, I want to conduct an in-person co-development research exercise with PPI partners to design the public survey about people's understanding of sustainable clinical research.

Why have you been invited to take part?

You have been invited to take part in the co-development of the survey because you are someone who may have been involved in clinical research in the past or have an interest in clinical research or the environment.

How will your data be used?

You will be asked to provide your name and contact information if you agree to take part. This will include your email address and/or your phone number. Information on your occupation or background may be collected to provide context for your involvement in the co-development aspect of this research study. Your details, including name, email address, occupation/background and/or phone number will be securely stored on the UCD network until the study is complete. Once the study is complete, your identifiable data (name, email address and/or phone number) will be destroyed in line with data protection guidelines. Information from the co-development exercise may be published in an academic journal or presented at scientific meetings, including conferences. No personally identifiable information will be published. A signed copy of this information leaflet and consent form will be provided to you if you agree to take part.

What will happen if you decide to take part in this co-development exercise?

If you agree to take part, you will be asked to agree to a set of statements and sign a consent form which is included in this document. You will be invited to attend two in-person co-development workshop meetings (each will be two hours) with two researchers and

other PPI partners. Both meetings will be held in person and recorded and transcribed so that the researchers can evaluate and write about the co-development process:

- At the first meeting, the lead researcher will present the survey topic and suggested themes to the PPI partners. You will receive a copy of the themes in advance of the meeting. The themes will be reviewed together at the meeting. Any edits to the themes and suggestions of new/alternative themes will be discussed.
- When the set of themes are approved, suggested questions and formatting of questions will be generated for each theme.
- The lead researcher will then draft a survey with the suggested themes and questions.
- At the second meeting, the lead researcher will present the drafted survey, and the group will review, discuss and finalise the questions and formatting.
- Overall, we will also strive to ensure the survey is clear, written in lay language and accessible.

You can withdraw from the co-development aspect of this study at any time. You do not have to provide a reason.

How will your privacy be protected?

A small amount of personal data will be collected about you for the purpose of agreeing to take part in this study. The data will be stored on a secure UCD network and any personal data (names, email addresses etc.) will be destroyed as soon as possible after collection. Any audio or written feedback you provide during the co-development exercise that may be deemed identifiable will be fully anonymised.

What are the benefits of taking part in this co-development exercise?

You may or may not benefit from taking part in this co-development exercise. It is hoped that you will gain valuable experience in this co-development research process. This includes developing skills, such as teamwork, active listening, content review, survey development, and accessibility awareness. It is also an opportunity to provide your voice and ensure that research is designed in a way that is relevant, understandable and impactful.

What are the benefits to the researcher if you take part in this co-development exercise?

The co-development exercise for this study is essential to ensure that the right questions are being asked, the survey is formatted and designed with the public in mind, and the language is simple and clear. The researcher acknowledges the importance of co-design with PPI partners and the immense benefit this brings to the research process. This exercise may also extend benefit to other researchers who may want to implement co-design aspects into their research processes.

What are the risks of taking part in this co-development exercise?

There will be minimal risk to you in taking part in this co-development exercise. You will provide your name for consent purposes and contact details for further contact purposes. This is known as personal data. Your personal data will be securely stored on a private drive within the UCD network and will be destroyed at the end of the study period. It is not anticipated that the co-development process will cause any distress or upset, however if you become distressed or upset and would like to stop taking part, please let the researcher know. You can withdraw from this co-development exercise at any time without needing to give a reason and your personal data, will be destroyed at that point if you wish.

Can you change your mind at any stage and withdraw from the co-development exercise?

Yes. You can change your mind and withdraw at any stage. If you would like to withdraw, please contact Mr Dylan Keegan at the details below. Your personal data will be destroyed.

How will you find out what happens with this project?

You will have the option of staying in contact with the researchers if you would like to find out what happens with this project. You are also welcome to reach out to me if you have any questions before, during, or after your participation in the co-development exercise.

Will I be paid for my time?

Yes. PPI partners will be remunerated for their involvement in the co-development exercise.

> Each meeting will be two hours long. You will be paid €22 per hour for these meetings, plus an additional 1 hour for preparation time and 1 hour for travel time. As such, you will be paid for **eight hours** in total for in-person attendance at two meetings. Travel expenses to attend any in-person meetings will be covered and refreshments will be provided.

Contact details for further information

If you have any questions about the research study, co-development exercise or your potential participation, or if you would like to withdraw from this at any stage, please contact Mr Dylan Keegan at dylan.keegan1@ucdconnect.ie.

Thank you for taking the time to read this information leaflet.

Public and Patient Involvement (PPI) Consent Form

I have read and understood the **Public and Patient Involvement (PPI) Information Leaflet** about this co-development research exercise. The information has been fully explained to me and I have been able to ask questions, all of which have been answered to my satisfaction.	**Yes** ☐	**No** ☐
I understand that I do not have to take part in this co-development research exercise and that I can opt out at any time. I understand that I don't have to give a reason for opting out and I understand that opting out won't affect me negatively in any way.	**Yes** ☐	**No** ☐
I am aware of the potential risks, benefits and alternatives of this co-development research exercise.	**Yes** ☐	**No** ☐
I give permission for researchers to securely store my personal data. I have been assured that my personal data will be kept private and confidential.	**Yes** ☐	**No** ☐
I give informed consent to have my data processed as part of this co-development research exercise.	**Yes** ☐	**No** ☐
I understand that the workshops will be audio recorded. The recordings will be securely stored until transcribed and destroyed thereafter. Identifiable information will not be included in any published materials.	**Yes** ☐	**No** ☐
I understand that information from this co-development research exercise that is published will not include identifiable information.	**Yes** ☐	**No** ☐
I consent to take part in this co-development research exercise having been fully informed of the risks, benefits and alternatives.	**Yes** ☐	**No** ☐

Participant Name   Date

---   ------------------------
