# Supplementary material for: Co-developing a survey on public understanding of sustainable clinical research: A study protocol
Source: PLoS One. 2026 May 11;21(5):e0342279. doi: 10.1371/journal.pone.0342279 (PMC13160332; doi:10.1371/journal.pone.0342279)
Supplement: S2 File — (PDF) [file pone.0342279.s002.pdf]

Participant Information Leaflet

Introduction

You are being invited to take part in this anonymous research study.

Before you decide whether to take part it is important for you to understand why this research is being done and what it will involve. Please take some time to read this participant information leaflet that will explain the purpose of this research study.

If you wish, talk to your friends and family about this research study. If there are any questions you have about the research study or if you are unclear about any of the information outlined in the information leaflet, please get in touch with Mr Dylan Keegan.

Research study topic: Sustainable clinical research
Research study title: To investigate public understanding of clinical research and its environmental impact
Researcher name: Mr Dylan Keegan
Researcher's school: School of Medicine, University College Dublin

What is this research about?

This research is about public understanding of clinical research. This includes environmental concerns that can be associated with carrying out clinical research. The research for this study will inform aspects of an overall PhD research project investigating sustainable clinical research.

Why I am doing this research?

This study is part of my PhD research project which is about sustainable clinical research. I want to learn more about public understanding of clinical research, including carrying out environmentally friendly research.

Why have you been invited to take part?

You have been invited to take part because you or someone you know may have been involved in clinical research in the past, or you may have an interest in clinical research.

How will your data be used?

This survey is anonymous. As such, no personal data will be collected about you. You will have the option of seeing and/or downloading a copy of the end-of-survey response report that will include a summary of your responses. If you would like to hear about the results of the study or if you have questions about the study, you may contact Mr Dylan Keegan at

dylan.keegan1@ucdconnect.ie. Your email address will be securely stored on a private UCD Network and destroyed in line with data protection guidelines once the study is completed. Results from this study may be published in an academic journal or presented at scientific meetings, including conferences. No personally identifiable information will be published.

<u>What will happen if you decide to take part in this research study?</u>

You will be invited to complete an anonymous survey. If you agree to take part, you will be asked to complete a set of questions across several themes related to clinical research. You can withdraw from the study by simply closing the survey **before** completing survey questions. You do not have to provide a reason. Once you have begun answering survey questions or the survey has been submitted, you will not be able to withdraw from the study, as no personal data will be collected about you and you will not be identifiable.

<u>How will your privacy be protected?</u>

The anonymous survey for this study will be hosted on a secure, UCD approved, survey platform, called Qualtrics. The data will be stored on a secure UCD network. No personal data will be collected about you. If you reach out to the researcher by email for further information about the study or if you require assistance, this data will be securely stored and destroyed as soon as possible after collection. Qualtrics has the option to anonymise responses, which disables the collection of personally identifiable information (PII), including IP addresses and contact information. This will be enabled if you complete the survey. Any written feedback you provide during the survey that may be deemed identifiable will be fully anonymised.

<u>What are the benefits of taking part in this research study?</u>

There will likely not be any benefit for you taking part in this research study.

<u>What are the benefits to the researcher if you take part in this study?</u>

The information gathered from this study will help me to understand more about people's knowledge about clinical research and environmental concerns about research. This may help in the future to design more environmentally friendly research and will help the researcher to complete parts of his PhD research.

<u>What are the risks of taking part in this research study?</u>

There will be minimal risk to you in taking part in this research study. This is an anonymous survey that will not collect personal data about you. If you provide your email address, it will be securely stored on a private drive within the UCD network and will be destroyed at the end of the study period. It is not anticipated that the survey questions will cause any distress or upset, however if you become distressed or upset and would like to stop taking part, please simply close the survey. Nothing further is required from you as your data is

anonymous. You can withdraw from this study by closing the survey **before** completing any questions. You may also close the survey after partially completing it. Your responses will be saved.

Can you change your mind at any stage and withdraw from the study?

Yes. You can change your mind and withdraw from the study by simply closing the survey **before** completing any questions. If you have completed or partially completed the survey, your responses cannot be identified, and it will not be possible to withdraw those responses.

How will you find out what happens with this project?

You are welcome to reach out to me if you have any questions before, during, or after completing the survey.

Contact details for further information

If you have any questions about the research study and your potential participation, please contact Mr Dylan Keegan at dylan.keegan1@ucdconnect.ie.

Thank you for taking the time to read this participant information leaflet.

Participant Consent Form

I have read and understood the **Participant Information Leaflet** about this research project. The information has been fully explained to me and I have been able to ask questions, all of which have been answered to my satisfaction.	**Yes □**	**No □**
I understand that I do not have to take part in this study and that I can opt out at any time. I understand that I don't have to give a reason for opting out and I understand that opting out won't affect me negatively in any way.	**Yes □**	**No □**
I am aware of the potential risks, benefits and alternatives of this research study.	**Yes □**	**No □**
I am over the age of 18 and I give permission for researchers to securely store personal data I may provide. I have been assured that any personal data provided will be kept private and confidential, and destroyed as soon as necessary.	**Yes □**	**No □**
I give informed consent to have my data processed as part of this research study.	**Yes □**	**No □**
I understand that information from this research that is published will not include identifiable information.	**Yes □**	**No □**
I consent to take part in this research study having been fully informed of the risks, benefits and alternatives.	**Yes □**	**No □**

Participant Name Date

--- ------------------------
