# Supplementary material for: Co-developing a survey on public understanding of sustainable clinical research: A study protocol
Source: PLoS One. 2026 May 11;21(5):e0342279. doi: 10.1371/journal.pone.0342279 (PMC13160332; doi:10.1371/journal.pone.0342279)
Supplement: Attachment — Submitted filename: Response to Reviewers.pdf [file pone.0342279.s005.pdf]

Editor and Reviewer Comments

Editor

We thank the editor for the additional information regarding journal requirements. The manuscript and files have been formatted to meet the journal's style requirements. This includes edits to the title page, citation formatting throughout, headings throughout, the reference list, and supporting information section.

Reviewer #1: This manuscript presents a well-designed and timely study protocol addressing the increasingly important issue of sustainability in clinical research. The integration of Public and Patient Involvement (PPI) in the co-development of the survey instrument is a notable methodological strength that enhances relevance, inclusivity, and rigor. The study design is clearly structured, ethically sound, and appropriately aligned with established qualitative and survey reporting frameworks. The manuscript would benefit from minor clarifications regarding the long-term data-sharing plan and a slightly expanded discussion on sampling limitations and policy implications. These refinements will improve transparency and strengthen the overall impact of the work. Subject to these minor revisions, the manuscript is suitable for publication.

We thank reviewer 1 for their valuable feedback. Clarification has been added regarding the long-term data-sharing plan under the heading 'Data management plan'. The discussion has been expanded to include points on limitations related specifically to the snowball sampling approach and how the authors will address this. Additionally, further detail regarding influence on policy implications has been added.

Reviewer #2: This manuscript presents a study protocol describing the co-development and deployment of a survey designed to evaluate public understanding of environmentally sustainable clinical research. The study integrates Public and Patient Involvement (PPI) through a structured co-development process and aims to generate insights into public awareness, perceptions, and attitudes toward sustainability in clinical research practices.

Overall, the protocol is clearly structured and well aligned with contemporary approaches to participatory research and sustainability in healthcare. The manuscript would benefit from minor clarifications and improvements in presentation prior to publication.

We thank reviewer 2 for their valuable feedback that has been addressed below.

Clarity of Study Rationale:
The introduction clearly outlines the environmental impact of healthcare and clinical research. However, the rationale for focusing specifically on public understanding could be strengthened by briefly explaining how such understanding may influence policy, research practices, or participation in sustainable research initiatives.

The introduction has been revised to include additional detail regarding the influence of public understanding on education initiatives, standardised environmental reporting and cost adjustments, as well as the potential influence on future participant recruitment and participation.

Description of Survey Instrument Development:
The manuscript describes the co-development process using two workshops with PPI partners. While the process is well explained, it may be helpful to briefly describe how the final survey will be structured (for example, approximate number of questions or estimated completion time) to give readers a clearer picture of the resulting instrument.

As the survey design is contingent on the co-development process it is unclear of the exact number of survey questions to be included. However, clarification has been added to note there are six potential themes for inclusion and we would expect to limit each theme to five questions. The estimated survey completion time should be 30 minutes. Once the survey has been finalised and pilot tested, the estimated completion will be updated and added to the survey brief.

Recruitment Strategy:
The protocol outlines several recruitment channels including clinical research networks, patient organisations, and social media distribution. A brief clarification of how duplicate responses or repeated participation will be prevented would improve methodological transparency.

This is an important consideration given the nature of the recruitment approach. A clarification has been added to note that a feature of the survey software can be enabled to prevent multiple submissions.

Bias Considerations:
The manuscript appropriately acknowledges potential survey biases such as response bias, sampling bias, and non-response bias. Expanding briefly on how snowball sampling may influence the representativeness of the sample would strengthen this section.

Clarification has been added regarding the challenge of achieving representativeness from a snowball sampling approach. The distribution method can be potentially limited, however the authors believe the chosen networks and communities on the distribution list will address this sufficiently.
